# Case report: Satralizumab therapy for bilateral refractory optic neuritis following the first dose of bivalent human papilloma virus vaccine

**DOI:** 10.3389/fimmu.2024.1499045

**Published:** 2024-11-19

**Authors:** Chuanbin Sun

**Affiliations:** Eye Center, Second Affiliated Hospital of Zhejiang University School of Medicine, Hangzhou, China

**Keywords:** human papilloma virus, bivalent HPV vaccine, optic neuritis, steroid pulse therapy, satralizumab

## Abstract

Demyelinating optic neuritis (DON) is a rare but sight-threatening ophthalmic condition which occasionally occurs after human papilloma virus (HPV) vaccination. We herein report a case of previously healthy 13-year-old girl who developed a bilateral refractory DON three days after the first dose of bivalent HPV vaccine. The patient experienced bilateral severe visual loss three days after HPV vaccination, and her vision was quickly deteriorated to no light perception one day after the onset of DON. Ophthalmic examination revealed sluggish pupillary light reflex and swollen optic disc in both eyes, and an emergent orbital MRI examination revealed bilateral hyperintensity and enlargement of the intraorbital optic nerve with contrast enhancement. Serological tests for aquaporin-4 IgG antibody, myelin oligodendrocyte glycoprotein IgG antibody, and other common autoantibodies were all negative. The patient showed poor response to 10 days of intravenous methylprednisolone pulse therapy (500 mg, 250 mg, and 125 mg twice per day for 4, 4, and 2 days, respectively). Hence, three-dosed subcutaneous satralizumab was used in the acute stage of DON as an adjunct therapy. Her vision gradually improved after satralizumab therapy, and increased to 20/20 and 20/32 in the right and left eye at the 3-month follow-up. To the best of our knowledge, this is the first case report of satralizumab therapy in the AQP-4 Ab and MOG-Ab dual seronegative isolated DON. Our study indicates that satralizumab may be a safe and efficient adjunct therapy which can be used in the acute stage of the refractory DON poorly responding to steroid pulse therapy.

## Introduction

Human papilloma virus (HPV) vaccines including a bivalent vaccine (HPV2), a quadrivalent HPV (HPV4), and a nine-valent (HPV9) are worldwidely used in adolescent girls and adult women to prevent HPV infection and subsequent cervical cancer (1, 2). Although the safety of HPV vaccines has been proved by several large-scaled clinical studies, serious adverse events including central neural system (CNS) autoimmune inflammations after HPV vaccination are occasionally reported (1–5), which give rise to a public concern on the safety of HPV vaccines.

Optic neuritis (ON) is a rare but sight-threatening ophthalmic condition which occasionally occurs in the infectious, post-infection, or post-vaccination cases. It is reported that demyelinating optic neuritis (DON) is the most predominant clinical manifestation of CNS autoimmune inflammations in cases after vaccination. ^1-5 A^ survey study based on the Regional Health Care Information Platform in Ningbo, China revealed an incidence of 1.8% (346/19328 cases) for DON in females receiving HPV4 or HPV2 vaccination (6). However, our literature research based on Pubmed database reveals that detailed clinical characteristics and natural course of DON after HPV vaccination are currently reported in only several cases (7–9).

Although most DON cases after vaccination show good response to steroid pulse therapy (7, 8, 10), there still exist a few cases who poorly respond to steroid pulse therapy and need other adjunct therapy such as plasma exchange to rescue their vision (9, 10). We herein report a case of a previously healthy girl who developed a bilateral refractory DON three days after the first dose of HPV2, satralizumab is used as an adjunct therapy in the acute stage of DON in this case due to her poor response to steroid pulse therapy.

## Patient presentation

**Figure 1:**
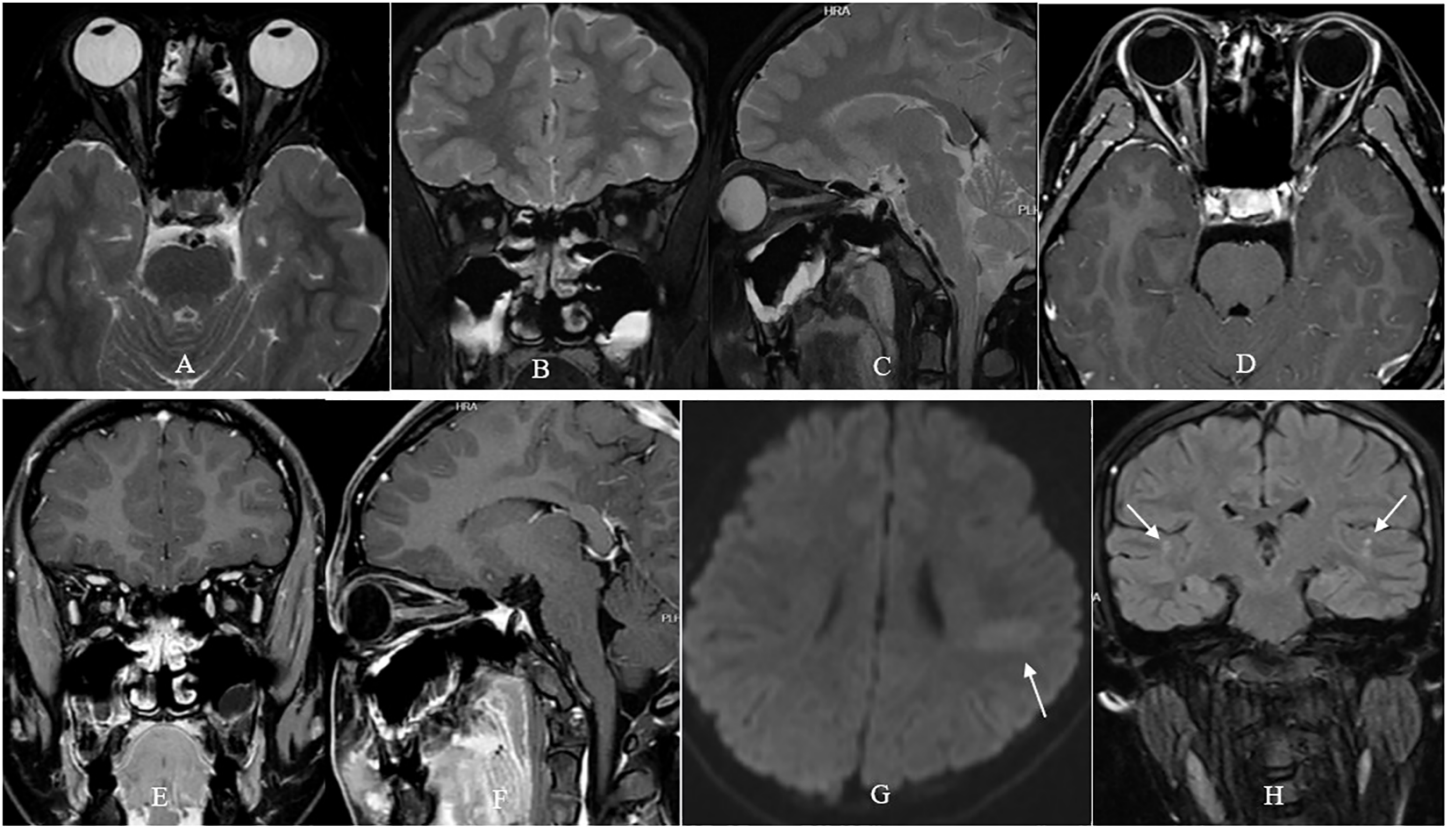
Orbital MRI examination of the patient. T2- fat suppression sequence showed long-segment hyperintensity and enlargement of the intraorbital optic nerve in both eyes **(A–C)**. Contrast-enhanced T1- fat suppression sequence showed long-segment contrast-enhancement of the intraorbital optic nerve in both eyes **(D–F)**. Fluid attenuated inversion recovery sequence showed an isolated finger-like slightly hyperintense lesion vertical to left lateral ventricle [arrow, **(G)**], and several spotty hyperintense lesions in bilateral parietal lobes [arrow, **(H)**].

A 13-year-old previously healthy girl was taken to the local hospital by her patients due to severe visual loss in both eyes accompanied by ocular pain when moving her eyes for four days. Initial ophthalmic examination showed sluggish pupillary light reflex and swollen optic disc in both eyes, and an emergent orbital MRI examination revealed long-segment hyperintensity and enlargement of the intraorbital optic nerve with contrast enhancement in both eyes (**Figures 1A–F**), as well as an isolated finger-like slightly hyperintense lesion vertical to left lateral ventricle (**Figure 1G**), and several spotty hyperintense subcortical lesions in bilateral parietal lobes (**Figure 1H**). Her post medical record was unremarkable. However, the girl just received the first dose of HPV2 (Yuxi Zerun Biotechnology Company, China) three days before her visual loss. The girl was diagnosed as bilateral DON and received 500mg of methylprednisolone intravenous injection per day for 3 days. Unfortunately, her vision in both eyes continuously deteriorated to no light perception one day after the initial steroid therapy, and got no improvement after 3 days’ steroid pulse therapy.

**Figure 2:**
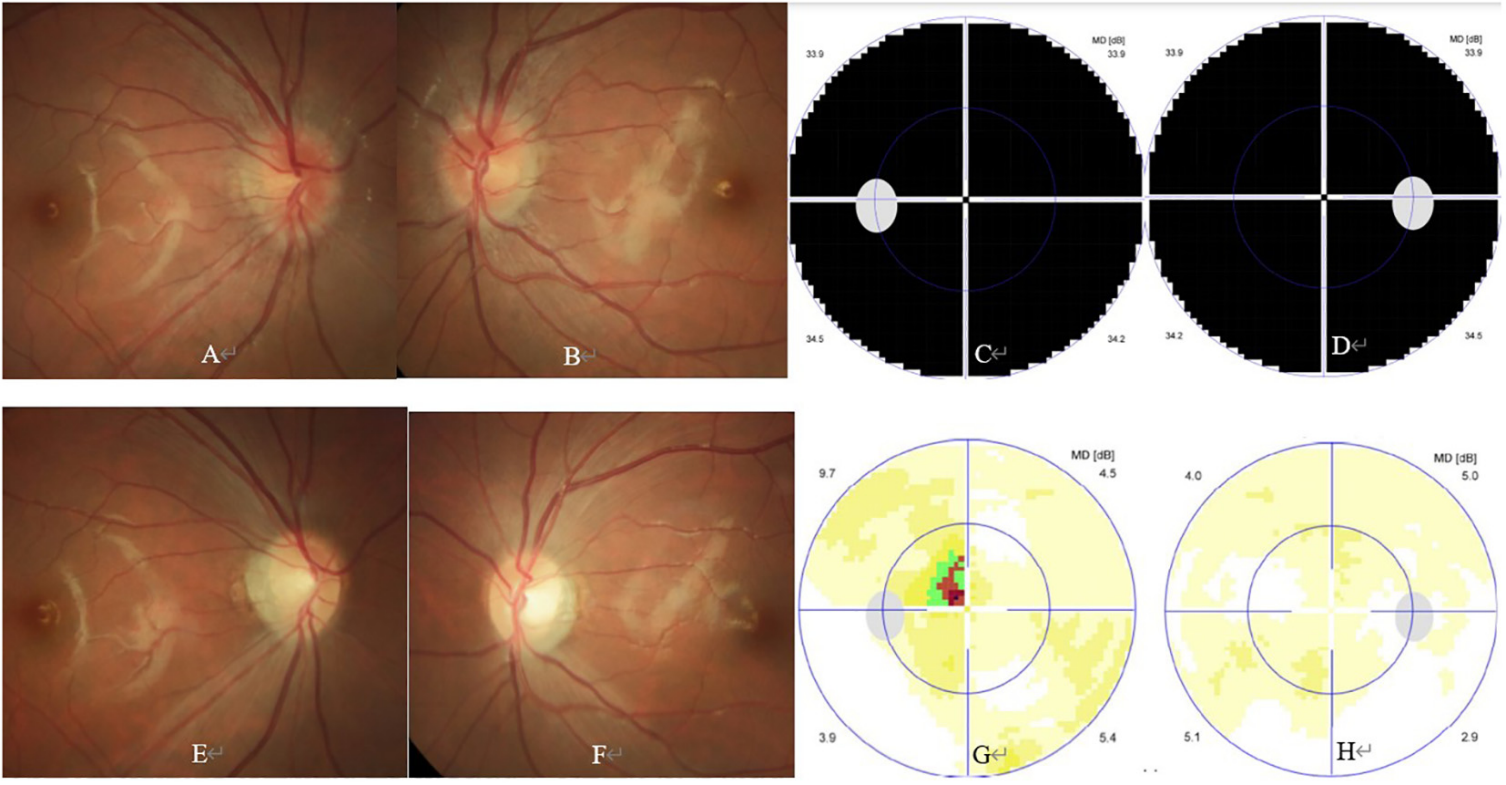
Ophthalmic and paraophthalmic examinations of the patient. At presentation, ophthalmic examination showed bilateral swollen optic disc **(A, B)**. Visual field test showed diffuse depression in both eyes **(C, D)**. At the 3-month follow-up, ophthalmic examination showed bilateral temporal optic disc pallor **(E, F)**. Visual field test showed small central scotoma in the left eye **(G)**, and normal result in the right eye **(H)**.

The girl was then referred to our Neuro-ophthalmology clinic for further consultation. At presentation in our hospital, ophthalmic examination revealed no light perception in both eyes, and clear cornea, quiet anterior chamber, dilated pupil of 8 mm and absent pupillary light reflex, clear lens and vitreous, and swollen optic disc in both eyes (**Figures 2A, B**). Visual field test showed diffuse depression in both eyes (**Figures 2C, D**). Spectral-domain optical coherence tomography (SD-OCT) revealed an average ganglion cell complex thickness of 99 μm in both eyes. Serological tests including antinuclear antibodies, antiphospholipid antibodies, antineutrophil cytoplasmic antibodies, and angiotensin converting enzyme were all negative. Commercial serological tests for aquaporin-4 IgG antibody (AQP-4 Ab) and myelin oligodendrocyte glycoprotein IgG antibody (MOG-Ab) by cell-based indirect dual-immunofluorescence assay were both negative. Microbiological screening tests for treponema pallidum, tubercle bacillus, hepatitis B virus, hepatitis C virus, human immunodeficiency virus, cytomegalovirus, rubella virus, herpes viruses and toxoplasma were all negative.

The diagnosis of bilateral DON was confirmed. The patient was then admitted into hospital, and intravenous injection of methylprednisolone 500 mg twice per day for 4 days, followed by 250 mg twice per day for 4 days, and then 125 mg twice per day for 2 days, was prescribed. Her visual acuity slowly improved from no light perception in both eyes to hand motion in the right eye and finger counting in the left eye respectively 3 days after admission, but kept stable in the following 7 days. Plasma exchange or immunoadsorption therapy was then highly recommended to rescue her vision, but was declined by the patient and her parents. Intravenous immunoglobulin injection was also refused by the patient and her parents when they were informed that immunoglobulin therapy might not help improving her visual function (11, 12).

After detailed communications with the patient and her parents on the severity of her ocular condition, and other alternative therapy especially the off-label use of monoclonal antibody therapy such as rituximab, ofatumumab, inebilizumab, and satralizumab, as well as their advantages and disadvantages, the patient and her patients finally chose satralizumab as an acute-staged adjunct therapy for this refractory DON. Ten days after her admission (2 weeks after the onset of DON), when the last dose of intravenous injection of methylprednisolone (125 mg) was completed, the girl received the first dose injection of satralizumab 120 mg subcutaneously, no side effects were reported after satralizumab injection. The girl was then discharged with a prescription of oral methylprednisolone 40 mg per day which was slowly tapering. The second and third dose of satralizumab was uneventfully injected 2 and 4 weeks after its initial injection, respectively.

At the 2-week, 4-week, and 3-month follow-up, the general condition of the girl was unremarkable, and her visual acuity gradually improved to 20/400 and 20/400; 20/40 and 20/67, and 20/20 and 20/32 in the right and left eye, respectively. At the 3-month follow-up, ophthalmic examination revealed normal anterior segment and temporal optic disc pallor in both eyes (**Figures 2E, F**). Visual field test showed normal result and small central scotoma in the right and left eye, respectively (**Figures 2G, H**). SD-OCT revealed an average ganglion cell complex thickness of 65 μm in the right eye and 61 μm in the left eye, respectively. The patient is still under follow-up, but receiving neither steroid nor satralizumab therapy now.

## Discussion

DON is a rare but sight-threatening ocular condition after vaccination. Many vaccines such as influenza, HPV, or severe acute respiratory syndrome coronavirus 2 (SARS-COV-2) vaccine were reported to be related to DON occurrence (1–10). Although many clinical studies have reported that vaccination does not increase the risk of DON occurrence, the temporal relationship between vaccination and the onset of DON indicates that vaccine may be a trigger of an initial onset or a relapse of DON (1–6, 10).

DON can occur isolatedly or as an ocular manifestation of CNS autoimmune inflammations after vaccination. Chang et al. reported two episodes of DON which occurred 7 days and 3 days after the first and second HPV vaccination respectively in a 30-year-old woman. Serological tests after the second episode of DON revealed AQP-4 Ab positive in their case (7). DiMario et al. reported permanent bilateral visual loss and left hemiparesis which occurred 10 days after the second dose of HPV vaccination in a 16-year-old girl. Serological tests revealed AQP-4 and other autoantibodies all negative in above case (9).

As for our case, one ON attack and orbital MRI examination revealed bilateral optic nerve enlargement and enhancement, an isolated finger-like hyperintense lesion vertical to left lateral ventricle and several spotty hyperintense subcortical lesions which indicating multiple spacial attacks implied a diagnosis of multiple sclerosis (MS). However, according to 2017 McDonald criteria, there still need MRI findings indicating multiple temporal attacks or cerebrospinal fluid oligoclonal band positive test result, to make a diagnosis of definite MS. Unfortunately, this case lacked of MRI findings indicating multiple temporal attacks and refused lumbar puncture which meant that no cerebrospinal fluid test was performed. Hence, currently we still lack of enough evidence to make a diagnosis of definite MS-ON.

Moreover, the case in this study showed severe bilateral ON (no light perception in both eyes) and poor response to two rounds of steroid pulse therapy, which was not consistent to a typical MS-ON manifestation (typical MS-ON manifested as unilateral optic neuritis, mild visual loss and good response to steroid therapy), but to an AQP-4 Ab negative neuromyelitis optica spectrum disorder (NMOSD)-ON. Hence, long-term follow-up is needed to further evaluate it is an isolated DON, or MS-ON, or even an AQP-4 Ab negative NMOSD-ON.

Currently, the exact pathogenesis of post-vaccination DON is still not clear. Possible mechanisms include the immediate immune disturbance and subsequent inflammatory cytokines release after vaccination, as well as the production of autoimmune antibodies including AQP-4 Ab and MOG-Ab induced by viral or bacterial vaccines via molecular mimicking or random exposure of antigens during inflammation after vaccination (7–10, 13, 14). Previous investigations have revealed that HPV vaccination can induce AQP-4 Ab positive or AQP-4 Ab and MOG-Ab double negative DON (7–10). Considering that DON in this case occurred only 3 days after the first dose of HPV2 vaccination, it is reasonable to speculate that DON in this case was probably caused by an immediate immune disturbance and subsequent inflammatory cytokines release after HPV vaccination, since autoimmune antibodies such as AQP-4 Ab or MOG-Ab cannot be produced within 7 days after vaccination (13, 14). This hypothesis is supported by the negative serological test results for AQP-4 Ab, MOG-Ab, and other common autoantibodies in this study.

Although most post-vaccination DON shows good response to steroid pulse therapy, there exist a few case reports indicating that some AQP-4 Ab seropositive or AQP-4 Ab and MOG-Ab dual seronegative cases showed poor or no response to steroid pulse therapy, and needed other adjunct therapies such as plasma exchange or immunoadsorption to rescue their vision (7–10). However, if plasma exchange or immunoadsorption therapy is not available, for example, because of plasma shortage, unaffordable expenses, or allergic reaction, monoclonal antibody such as rituximab and satralizumab is an alternative for adjunct therapy for the refractory DON and NMOSD (15, 16).

Interleukin-6 (IL-6) is one of the most important pro-inflammatory cytokines, and can facilitate the production of the acute-phase inflammatory proteins, the maturation of B cells and production of antibodies, as well as the maturation of cytotoxic T cells. It is reported that IL-6 plays an important role in the pathogenesis of NMOSD, and several studies revealed that serum IL-6 level significantly increased in NMOSD cases during relapse, and that cerebrospinal fluid IL-6 level significantly increased in an acute disseminated encephalomyelitis case at its initial onset (17, 18). Satralizumab is a humanized monoclonal antibody specific for IL-6 receptor, and has been approved to significantly reduce the relapse rate in AQP4-Ab positive NMOSD cases in several clinical trials (15, 16, 19). However, the efficiency of satralizumab therapy in AQP4- Ab seronegative NMOSD cases is still under discussion. A phase 3 clinical trial revealed that satralizumab monotherapy could not decrease the relapse rate in AQP4- Ab seronegative NMOSD cases (19).

To the best of our knowledge, this is the first case report of satralizumab therapy for the AQP-4 Ab and MOG-Ab dual seronegative isolated DON. Findings in this case indicate that satralizumab may be a safe and efficient adjunct therapy which can be used in the acute stage of the refractory DON which shows poor or no response to steroid pulse therapy, even though the DON is AQP-4 Ab and MOG-Ab dual seronegative. However, a large-scaled randomized control study is needed to further evaluate the necessity and efficacy of satralizumab in acute-staged DON therapy.

## Data Availability

The original contributions presented in the study are included in the article/supplementary material. Further inquiries can be directed to the corresponding author.
